# Reliability of Diagnostic Tests for *Helicobacter pylori* Infection

**DOI:** 10.1155/2011/940650

**Published:** 2011-08-01

**Authors:** S. Redéen, F. Petersson, E. Törnkrantz, H. Levander, E. Mårdh, K. Borch

**Affiliations:** ^1^Division of Surgery, Department of Clinical and Experimental Medicine, Faculty of Health Sciences, University Hospital, Linköping University, 581 85 Linköping, Sweden; ^2^Department of Surgery, County Council of Östergötland, 581 85 Linköping, Sweden; ^3^Department of Pathology, National University Health System, 119074, Singapore

## Abstract

*Introduction*. *Helicobacter pylori (H. pylori)* infection is very common worldwide. A reliable diagnosis is crucial for patients with *H. pylori*-related diseases. At followup, it is important to confirm that eradication therapy has been successful. There is no established gold standard for the diagnosis of *H. pylori* infection. *Material and Methods*. A sample of 304 volunteers from the general population was screened for *H. pylori* infection with serology, ^13^C-urea breath test (UBT), rapid urease test (RUT) on fresh biopsy, culture from biopsy, and histological examination. Culture was used as gold standard. *Results*. The sensitivity was 0.99 for serology, 0.90 for UBT, 0.90 for RUT, and 0.90 for histological examination. Corresponding specificities were 0.82, 0.99, 0.98, and 0.97, respectively. The accuracy was 0.86 for serology, 0.96 for UBT, 0.95 for RUT, 0.93 for culture, and 0.95 for histology. There was a strong correlation between the results of UBT and the histological scores of *H. pylori* colonisation as well as between the results of UBT and the scores of RUT. *Conclusion*. There were only minor differences in accuracy between the three invasive tests for *H. pylori* infection in this population. RUT may be recommended as the first choice since a result is obtained within hours. The accuracy of UBT was comparable to the invasive tests, and it is recommended for situations when endoscopy is not needed.

## 1. Introduction

*Helicobacter pylori (H. pylori*) infection is very common worldwide [1–3]. The infection causes chronic gastritis which significantly increases the risk of developing gastric or duodenal ulcer, gastric adenocarcinoma, and mucosa-associated lymphoid tissue (MALT) lymphoma [2, 4]. 

Recommended indications for *H. pylori* eradication therapy are: ulcer disease, MALT lymphoma, atrophic gastritis, post gastric cancer partial resection, maintenance nonsteroidal anti-inflammatory drug treatment (NSAID) or aspirin ASA), and *H. pylori* infection in first degree relatives to gastric cancer patients [5, 6]. Bleeding ulcer, sometimes life threatening, is a common clinical consequence of *H. pylori* infection [7, 8]. The incidence of bleeding ulcer (gastric and duodenal) is almost unchanged since many years, although there are reports indicating that the incidence of duodenal ulcer is declining [8, 9]. Considering the fact that the fraction of NSAID- or ASA- (including low dose) related or idiopathic ulcers has increased, a correct aetiological diagnosis is mandatory in ulcer disease [10–12]. This further underlines the necessity of a reliable diagnosis of *H. pylori* infection both before and after eradication therapy [13–17]. 

Noninvasive clinical tests for detection of *H. pylori* infection are serology (e.g., IgG or IgA antibodies against cell-surface antigens), ^13^C-urea breath test (UBT), and faecal antigen tests [14, 15]. Serology mirrors past (within years) or current infection. The reported sensitivity and specificity of serology measuring IgG antibodies is 80–100% and 69–95%, respectively [14–16]. Reported sensitivity and specificity for UBT is 81–100% and 80–98%, respectively [14–16, 18, 19]. Differences between studies may in some instances be explained by differences in methodology and the choice of gold standard. In patients with bleeding peptic ulcer the performance of UBT seems to be superior to biopsy-based methods and histological examination seems to be superior to RUT [20]. 

Invasive tests for diagnosis of *H. pylori* infection are the rapid urease test (RUT), histological examination, and culture of gastric mucosal biopsies. Depending on the choice of gold standard, the sensitivity and specificity of RUT is 80–95% and 90–100%, respectively, [14–16, 18]. Histological examination has a sensitivity of 83–95% and a specificity of 90–100%, respectively [15, 16, 18]. For culture, the reported sensitivity and specificity is 80–90% and 95–100%, respectively [15, 16]. 

Considering the biopsy-based tests, the outcome probably is influenced by how many and where biopsies are taken both in the elective and acute (bleeding) situations [13, 20–23].

There seems to be no firm agreement as to which method should be used as gold standard for the detection of *H. pylori *infection. The aim of this study was to determine the concordance between, and accuracy of, five different tests for *H. pylori* infection in a population-based cohort examined with biopsies from both the antrum and corpus of the stomach. Culture was used as gold standard.

## 2. Material and Methods

### 2.1. Study Population

The prevalence and natural history of chronic gastritis and *H. pylori* infection in the studied cohort have been published [24, 25]. The participants were initially randomly selected from the population register of the mixed municipality of Linköping, Sweden. In association with the follow-up study [25], the occurrence of *H. pylori* infection was tested with five different methods (serology, UBT, RUT, culture, and histology) in 304 out of 314 participants. None of the *H. pylori* infected participants had received eradication therapy. Ten participants had subclinical prepyloric or duodenal ulcer at endoscopic screening [25].

### 2.2. Endoscopy

The volunteers fasted for at least six hours before EGD. Blood samples were drawn and EGD carried out after pharyngeal anaesthesia with lidocaine spray (Xylocaine, AstraZeneca, Södertälje, Sweden). Sedation with 2-3 mg intravenous flunitrazepam (Dormicum, Roche AB, Stockholm, Sweden) was given on demand. Three biopsy specimens were routinely collected from the gastric body (major, anterior and posterior aspect) and antrum (within 3 cm of the pylorus) for histological classification of chronic gastritis, including grading of *H. pylori* infection, according to the revised Sydney system [26]. One additional biopsy specimen from each of the corpus and antrum was collected for culture of *H. pylori*, and further one fresh biopsy from each location was analyzed for *H. pylori* with RUT (CLO-test, Delta West Pty Ltd, Bently, Australia).

### 2.3. Diagnostic Tests

Blood samples were stored at −80°C until analyzed. Serum IgG antibodies to *H. pylori* surface antigens were analyzed by ELISA as previously described, and results are given as relative optical density (OD), that is, in percent of positive standards (upper normal limit 5%) [24, 27]. 

^13^CO_2_-UBT was performed after fasting as in clinical routine in a VG ISOCHROM-*μ*G mass spectrometer (Fisons, UK). Breath samples were taken before and 15, 30, 45, and 60 min after ingestion of 50 mg ^13^C urea. The result used is from the 30-minute plot on the curve (delta over baseline 30) with an upper limit of 3.5 per mille. The participants were fasting and instructed to avoid proton pump inhibitors (PPI) two weeks before the test. Those in need of PPI, for example, for gastroesophageal reflux disease, were prescribed low dose H2-blockers during the two weeks preceding UBT. 

After orientation, fixation in neutral formaldehyde, and routine processing of the gastric biopsies, sections cut (5-*μ*m thick) perpendicular to the surface were stained with hematoxylineosin, alcian blue-periodic acid-Schiff, and Giemsa stain. The histological degree of *H. pylori* colonisation in biopsy sections was scored as none, mild, moderate, or severe (0, 1, 2, 3). 

The microscopic examinations were performed by an experienced pathologist without knowledge of other data. Kappa analysis of blinded repeat evaluation of the degree of *H. pylori* colonisation in biopsy sections from the antrum and corpus in 50 participants (20 without gastritis or *H. pylori* infection and 30 with chronic gastritis of whom 22 had *H. pylori* infection) yielded a Cohen's Kappa statistic of 0.897 and 0.824, respectively.

Frozen biopsies kept at −80°C in glycerol containing freeze medium were defrosted in room temperature, homogenized, and spread onto *H. pylori* selective agar plates (developed at the Microbiology laboratory (LMC), University Hospital of Linköping, Sweden). One culture medium was used. This is specific for *H. pylori* and contains GC agar (Acumedia, UK) developed at the accredited Microbiology laboratory (LMC), University Hospital of Linköping, Sweden. The bacteria were cultured under microaerophilic conditions at 37°C and read after 5–7 days. Translucent colonies typical for *H. pylori* were recultured and read after another 5–7 days. After another seven days urease, catalase, and oxidase tests were done to confirm that the colonies were *H. pylori,* all three tests should be positive. 

One fresh biopsy was taken from the corpus and antrum and tested for occurrence of *H. pylori* with RUT (CLO-test, Delta West Pty Ltd, Bentley, Australia), which was read after 20 min and 1, 3, and 12 h (scored 4,3,2,1, resp.). Absence of *H. pylori* according to RUT was scored 0.

### 2.4. Statistics

Agreement between the results of the *H. pylori* tests was evaluated by calculating the Cohen's kappa coefficient. Dichotomized data were used to calculate sensitivity, specificity, positive predictive value (PPV), negative predictive value (NPV), and accuracy. The values are given with 95% confidence interval. Each method was tested against culture as gold standard. The Kruskal-Wallis test was used to compare results of UBT between the histological scores of *H. pylori* colonisation and the scores of RUT, respectively. In all analyses, a two-sided *P* value of less than 0.05 was regarded as significant.

## 3. Results

The median age of the 304 participants of whom 143 were women was 66.1 (45.3–87.9) years. The results of the different tests for *H. pylori* infection are presented in Table 1. Of all 304 participants approximately 1/3 had current infection.  Table 2 shows the Cohen's kappa coefficients for agreement between the diagnostic methods. The best agreement was between RUT and culture (0.90) and between UBT and culture (0.91), compartment of the stomach was disregarded. 

Results of comparisons between the different tests for *H. pylori* infection are presented in Table 3. Considering the noninvasive tests (Table 3(a)), the UBT showed best accuracy at 0.96 (0.93–0.98), whereas the corresponding value for serology was 0.86 (0.82–0.90). Among the invasive tests, location in the stomach disregarded, accuracy ranged between 0.93 and 0.95. The invasive tests showed slightly better accuracy in the antrum than in the corpus.

**Figure 1:**
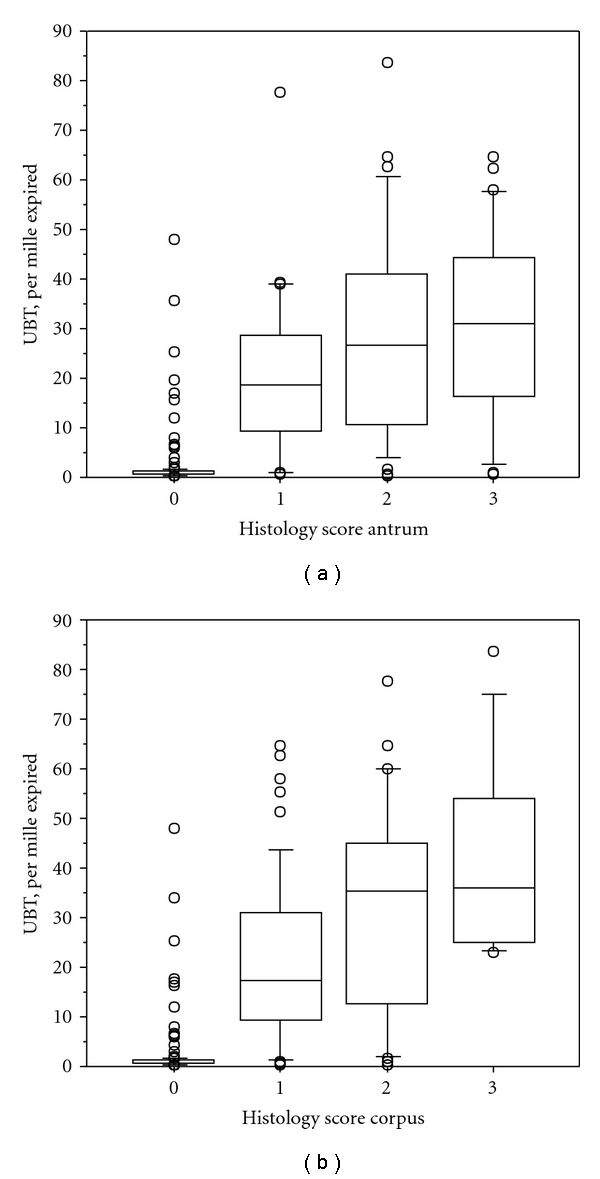
Boxplots (showing median and interquartile ranges) of the relation between UBT (per mille) and histological score of *H. pylori* colonisation.

**Figure 2:**
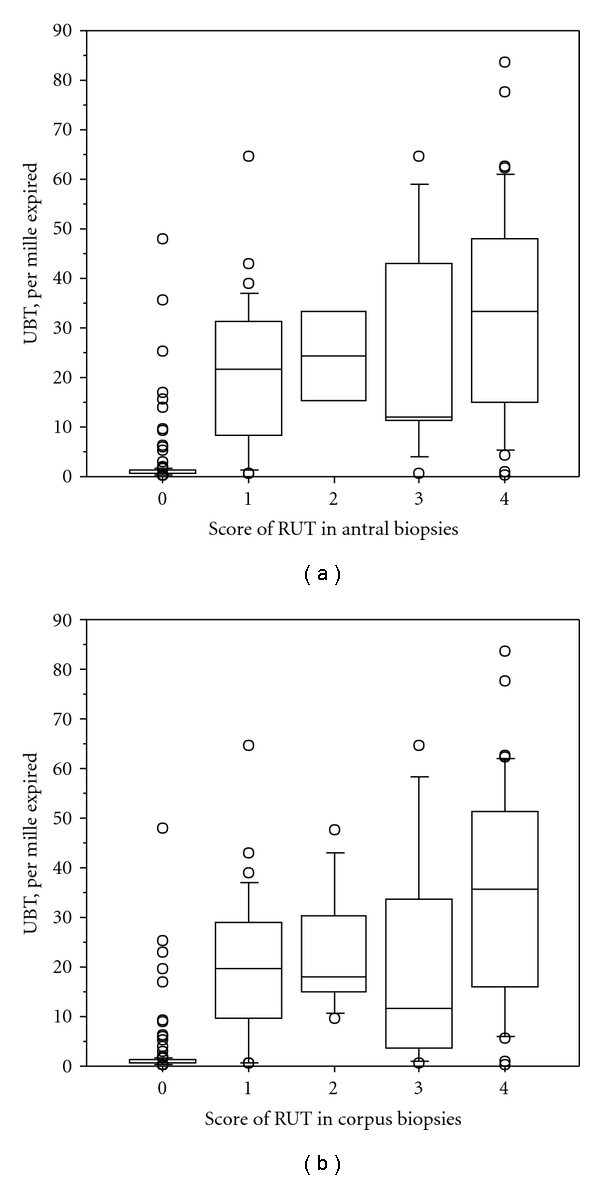
Boxplots (showing median and interquartile ranges) of relation between RUT score and histological score of *H. pylori* colonisation.

Relations between the results of UBT and histological scores of *H. pylori* colonisation in the corpus and antrum are illustrated in Figure 1. The two variables were strongly correlated considering both the antrum and corpus (*P* < 0.001, Kruskal-Wallis test). A similar strong correlation for both the antrum and corpus was present when the results of UBT were related to the scores of RUT (*P* < 0.001) (Figure 2).

## 4. Discussion

A reliable primary diagnosis and control of treatment success of *H. pylori* infection is crucial for patients with uncomplicated or complicated ulcer disease, MALT lymphoma, atrophic gastritis, previous partial gastric resection for gastric cancer, and probably also for *H. pylori* infected patients starting long-term medication with NSAID or low dose ASA [6, 28]. 

The aim of this study was to compare the results of five different* H. pylori* infection tests in a population-based cohort. These were serology, UBT, RUT, culture, and histological examination. Regrettably, stool antigen tests for *H. pylori* were not available when subjects were included to this study. Concordance between the tests according to Cohen's kappa analysis was calculated and we used sensitivity, specificity, PPV (precision), NPV, and accuracy to evaluate which combination of the tests may be recommended. 

Considering the invasive tests, potential sources of error, are that too few gastric biopsies, are analyzed and that both main compartments of the stomach are not represented [13, 17]. In the present study, three biopsies from each location were analyzed histologically. Although, Warthin-Starry stain for *H. pylori* may be more sensitive than giemsa, it is a cumbersome stain to perform. Moreover, the pathologist (FP) was used, from clinical practice, to evaluate *H. pylori *status based on giemsa stained sections.

According to Cohen's kappa analysis, the intraexaminator error for histological diagnosis of *H. pylori* colonisation was low. RUT and culture, respectively, were performed on one biopsy from each compartment. Collection of more than one biopsy from each location for these tests could potentially have influenced the results. A potential error in the UBT is use of PPI prior to testing. The study participants were instructed to avoid PPI two weeks before the examination. Participants on PPI medication, for example, for gastroesophageal reflux disease, were prescribed low dose H2 blockers during the two weeks preceding UBT. 

Agreement between the tests according to Cohen's kappa analysis was good (0.91) for culture (compartment of the stomach disregarded) and UBT. The result was similar for RUT and culture (0.90). The agreement between the two latter was betters in the antrum (0.90) than in the corpus (0.84). 

We chose to use accuracy as a measure of the performance of the diagnostic tests. Of the two noninvasive tests, UBT showed the highest accuracy (0.96 versus 0.86 for serology). Considering the invasive tests, results were similar; RUT (0.95), culture (0.93), and histology (0.95). The accuracy of the invasive tests was slightly lower (0.90–0.92) in the corpus compared with the antrum (0.93–0.96). We found no studies reporting accuracy of the diagnostic tests. 

In this study, the lowest sensitivity was for UBT (0.89) and the highest for serology and culture (0.99). Considering the specificities, the lowest was for serology (0.82) and the highest was for UBT (0.99). PPV was lowest for serology (0.66) and highest for UBT (0.99), whereas NPV only differed slightly between the tests (0.95–0.99). 

In a study by Cutler et al. [14], using several tests taken together as gold standard, sensitivity, specificity, PPV, and NPV were calculated for ^13^C UBT, serology, RUT, microscopic occurrence of *H. pylori, *and chronic and acute gastritis. Considering the first four tests, differences in results between that study and the present were minor regarding sensitivities and specificities, whereas differences were greater for predictive values, that is, PPVs were lower (UBT 0.99 versus 0.98, RUT 0.96 versus 1.0, serology 0.66 versus 0.95, and histology 0.94 versus 0.99) and NPVs higher in the present study. This finding may be related to differences between the studies with regard to the number and location of biopsies collected and that chronic gastritis was included among the diagnostic methods in the referred study [14]. Furthermore, there was a difference in the administered dose (150 mg versus 50 mg) of urea and the time interval (60 min versus 30 min) until the reading of the UBT. Another difference between the studies is that the referred one concerns patients, whereas the present one concerns a population-based cohort.

In a study from 2009 by Calvet et al. including 118 patients and using a predefined gold standard (more than one positive test result), the performance of ^13^C UBT, RUT, microscopic examination, and fecal tests was evaluated [18]. The PPVs found in that study were somewhat higher than those in the present study (RUT 1.0 versus 0.83, histology 0.99 versus 0.81, and UBT 0.92 versus 0.87). These differences may partly be explained by the fact that only antral biopsies were examined in that study, whereas both compartments of the stomach were examined in the present one. 

Average values of sensitivities and specificities of invasive and noninvasive tests were calculated in an overview of epidemiology and diagnosis of *H. pylori* infection by Logan and Walker [15]. Our results were within the range of these average values. The sensitivities and specificities of microscopically examination ranged between 88–95% and 90–95%, respectively. Corresponding values for culture were 80–90%, 95–100%, 90–95%, and 90–95% for the urease test. For serology, the sensitivities were 80–95% and the specificities 80–95%. Sensitivities and specificities for ^13^C-UBT ranged between 90–95% and 90–95%, respectively. Culture was mentioned as the theoretical gold standard. 

In a review by Chey and Wong [5], in guidelines of the American College of Gastroenterology, the urease test showed sensitivities of more than 90% and specificities of more than 95%. Corresponding values for histology were more than 95% and more than 95%. Considering serology, sensitivities ranged between 76% and 84% and the specificities between 79% and 90%, respectively. For UBT, both the sensitivities and the specificities were higher than 95%. Histological examination was used as gold standard. 

In conclusion, there were only minor differences in accuracy between the three invasive tests for *H. pylori *infection in this population. RUT may be recommended as the first choice since a result is obtained within hours. The accuracy of UBT was comparable to the invasive tests and it is recommended for situations when endoscopy is not necessary.

##  Ethics

The study was performed in accordance with the Helsinki Declaration and was approved by the local ethics committee. Informed written consent was obtained from all participants.

##  Conflict of Interests

The authors report no conflict of interests and have no financial interest to disclose.

## Figures and Tables

**Table 1:** Results of different tests for *H. pylori* infection in population-based cohort of 304 subjects.

Diagnostic method	Positive of all tested *N* (%)	Corpus and/or antrum, positive of all tested, *N* (%)	Antrum, positive of all tested *N* (%)	Corpus, positive of all tested, *N* (%)
Serology	119 (39.1)	—	—	—
UBT	91 (29.9)	—	—	—
RUT	—	95 (31.3)	88 (28.9)	89 (29.3)
Culture	—	101 (33.2)	91 (30.1) ^a^	98 (32.3) ^b^
Histology	—	97 (31.9)	86 (28.3)	89 (29.3)

UBT: ^13^C-urea breath test.

RUT: rapid urease test.

^a^no culture in two,  ^b^no culture in one.

**Table 2:** Agreement between the results of the tests for *H. pylori* as evaluated by the Cohen's kappa coefficient in study population of 304 subjects.

Test	Antrum and/or corpus	Antrum	Corpus
Serology-UBT	0.77	—	—
Serology-RUT	0.77	—	—
Serology-culture	0.84	—	—
Serology-histology	0.77	—	—
UBT-RUT	0.86	—	—
UBT-histology	0.83	—	—
UBT-culture	0.91	—	—
RUT-culture	0.90	0.90 ^a^	0.84 ^b^
RUT-histology	0.86	0.86	0.84
Culture-histology	0.88	0.82 ^a^	0.85 ^b^

RUT: rapid urease test, UBT: ^13^C-urea breath test.

^a^culture failed in two subjects,  ^b^culture failed in one subject.

**Table:** (a) Antrum and/or corpus. *N* = 304

Diagnostic test	Sensitivity (95% ci.)	Specificity (95% ci.)	PPV (95% ci.)	NPV (95% ci.)	Accuracy (95% ci.)
Serology	0.99 (0.93–1.00)	0.82 (0.76–0.87)	0.66 (0.56–0.74)	0.99 (0.97–1.00)	0.86 (0.82–0.90)
UBT	0.89 (0.81–0.94)	0.99 (0.97–1.00)	0.99 (0.94–1.00)	0.95 (0.91–0.97)	0.96 (0.93–0.98)
RUT	0.90 (0.83–0.95)	0.98 (0.95–0.99)	0.96 (0.90–0.99)	0.95 (0.91–0.98)	0.95 (0.92–0.97)
Histology	0.90 (0.83–0.95)	0.97 (0.94–0.99)	0.94 (0.87–0.98)	0.95 (0.91–0.98)	0.95 (0.92–0.97)

**Table:** (b) Antrum. *N* = 302 (culture failed/no growth in two subjects)

Diagnostic test	Sensitivity (95% ci.)	Specificity (95% ci.)	PPV (95% ci.)	NPV (95% ci.)	Accuracy (95% ci.)
RUT	0.91 (0.83–0.96)	0.98 (0.95–0.99)	0.95 (0.89–0.99)	0.96 (0.93–0.98)	0.96 (0.93–0.98)
Histology	0.85 (0.76–0.91)	0.96 (0.93–0.98)	0.91 (0.82–0.96)	0.94 (0.89–0.96)	0.93 (0.89–0.95)

**Table:** (c) Corpus. *N* = 303 (culture failed/no growth in one subject)

Diagnostic test	Sensitivity (95% ci.)	Specificity (95% ci.)	PPV (95% ci.)	NPV (95% ci.)	Accuracy (95% ci.)
RUT	0.94 (0.86–0.98)	0.92 (0.87–0.95)	0.78 (0.68–0.86)	0.98 (0.95–0.99)	0.92 (0.89–0.95)
Histology	0.94 (0.81–0.96)	0.90 (0.85–0.94)	0.74 (0.64–0.83)	0.96 (0.93–0.99)	0.90 (0.86–0.93)
